# Knowledge and attitudes toward euthanasia among final year pharmacy and law students: a cross-sectional study

**DOI:** 10.1186/s40545-023-00530-7

**Published:** 2023-03-09

**Authors:** Tidenek Mulugeta, Sintayehu Alemu

**Affiliations:** School of Pharmacy, Institute of Health, Jimma University, P. O. Box: 378, Jimma, Ethiopia

**Keywords:** Euthanasia, Law students, Pharmacy students, Jimma University

## Abstract

**Background:**

Euthanasia is the last resort for those living with untreatable and terminable diseases which cause pain and suffering. However, the concept of euthanasia resulted in many dilemmas and controversy around life extension and death.

**Objective:**

The aim of this study was to evaluate the knowledge and attitudes of final year pharmacy and law students concerning euthanasia.

**Methods:**

A descriptive cross-sectional study was carried out among all final year law and pharmacy undergraduate students. The data were collected using self-administered structured questionnaire and analyzed by SPSS version 22. Multivariate logistic regression was used to assess the influence of socio-demographic characteristics of participant’s on acceptance of euthanasia.

**Result:**

72 (61.5%) of the students were declared that euthanasia is administration of lethal drugs to a patient at the explicit request of that patient. Majority 87 (74.4%) of the students knew that euthanasia is active shortening of the dying process. Most participants 95(81.2%) awared that there is no legalized euthanasia in Ethiopia. On the other hand, 47(40.2%) believed the patient has the right to choose to end his/her own life. Around 45% had the view that euthanasia should be legalized in some circumstances. Only 27.3% (*n* = 32) of the respondents endorsed legalization of euthanasia in Ethiopia. 35 (29.9%) said euthanasia should be performed. The acceptance of euthanasia was greater for pharmacy students compared to law students [adjusted odds ratio (AOR) = 3.490; 95% CI 1.346–9.049; *p* = 0.010] and lower for Muslim students compared to Orthodox students (AOR = 0.186; 95% CI 0.044–0.783; *p* = 0.022).

**Conclusion:**

The final year law and pharmacy students were aware of euthanasia. However, majority of students did not reveal favorable attitude toward euthanasia and its acceptance was low. Participants’ field of study and religion were significantly affect acceptance of euthanasia As the current study limited to pharmacy and law students, the authors suggest that future studies should involve various segments of societies to investigate more about euthanasia in Ethiopia.

## Background

Since twentieth century, chronic and degenerative diseases have become more prevalent, replacing infectious diseases as the leading causes of mortality [1]. At the same time, the continuous biomedical advancements have also contributed to the life expectancy of these patients [2, 3]. But the extension of survival is frequently accompanied by discomfort and suffering from a variety of ailments. This prompted concerns about the efficacy of interventions meant to prolong life because they can cause a person to experience more misery and humiliation before passing away [1, 3].

The last resort for persons suffering from incurable, terminal illnesses that inflict agony and suffering is euthanasia [4, 5]. However, the idea of euthanasia gave rise to a number of problems about life extension and death. It is a contentious issue in a variety of contexts, including politics, society, philosophy, and religion, as well as in the fields of law and medicine [2, 6–8].

The goal of euthanasia is to end the life of a patient who has suffered prolonged, unrelenting, and intolerable suffering in order to keep him/her from experiencing further similar misery [9–14]. It is defined by modern medicine as an effort to prevent unnecessary and protracted suffering, taking into account the possibility that relieving pain or other symptoms could reduce someone’s lifespan [15] such as the administration of lethal drugs to a patient at the explicit request of that patient [10, 16, 17]. In practice, euthanasia can be passive or active. Active euthanasia is taking deliberate action to end the patient’s life. Passive euthanasia is refraining from action to keep the patient alive [8, 15, 18].

Currently, euthanasia legally practiced in the Netherlands, Belgium, Luxembourg, Colombia, and Canada [9–11]. On the other hand, it is prohibited in most countries of the world, including Ethiopia. Legalizing such a contentious activity necessitates the participation of numerous societal groups and professions, including law and pharmacy. Consequently, there is a valid justification for choosing pharmacy and law students as the research population. Students of pharmacy are important since their future careers may involve dispensing drugs for the goal of ending life, whereas students of law are obviously future decision-makers. Lawyers have a crucial role in end-of-life decision-making in country such as Belgium as they are involved in evaluating requests for euthanasia as members of the ethical committees. They focus on the legal and regulatory aspects of medical decisions while pharmacist along with other health care team might be involved in a curing, caring, and executive role and consider the perspective of the patient in the decision-making process [1].

In several researches, it has been discovered that lawyers have a favorable attitude about euthanasia [12] and that law students are more likely than medical students to accept euthanasia [19]. When pharmaceuticals are recommended for euthanasia, pharmacists are in a difficult situation and may need to evaluate if giving a patient a deadly amount of a prescription is morally and ethically appropriate [20]. Because of this moral implication, pharmacists have a responsibility to be interested in and informed about their patients' health outcomes. They are accountable for the moral distribution of medications, particularly those used for euthanasia. The establishment of extensive and strong health policies that assure the safe and appropriate use of medications, including those used in euthanasia is therefore made better by considering pharmacists' perspectives [11, 21].

Since pharmacy students represent the next generation of pharmacists, it is crucial for the healthcare team to understand their perspectives on euthanasia. Future pharmacists in Ethiopia might come into contact with patients who are suffering from terminal illnesses at some point in their employment; therefore it makes sense for them to be familiar with euthanasia. Regardless of whether euthanasia is made legal in Ethiopia, having this knowledge could be very helpful in discussions with patients (or their families) that may have questions about the end of life and euthanasia. To the best of our knowledge, investigations of a similar nature have not been carried out in Ethiopia, but they have in other nations. Therefore, this study  was aimed to evaluate the knowledge and attitudes of final year pharmacy and law students concerning euthanasia. The study also investigated the impact of socio-demographic characteristics on acceptance of euthanasia.

## Methods

### Study setting and period

The study was conducted in Jimma University at school of pharmacy and law. The University is located in Jimma town, situated around 346 km southwest of Addis Ababa, the capital of Ethiopia. The curricula of both pharmacy and law trainings at Jimma University take 5 years duration. During their 5-year stay, the students have enough exposures to formal class sessions relating to euthanasia. The study was conducted from January 12 to 30, 2022 at school of pharmacy and law Jimma University.

### Study design

A descriptive cross-sectional study was carried out among all final year law and pharmacy undergraduate students.

### Study participants and sampling procedure

The final year pharmacy and law undergraduate students currently enrolled at school of pharmacy and law were invited to participate in the study. Since there are limited numbers of final year students in both schools, no form of sampling was carried out and all volunteered students were included in this study. Students unwilling to participate were excluded from the study. Participation in the study was merely based on willingness of the participants without any incentive.

### Data collection instrument and techniques

A self-administered structured questionnaire was developed in English after intensive review of related published articles [1, 5, 8, 9, 12, 18, 19, 22, 23]. The questionnaire comprised questions pertaining to students' socio-demographic characteristics, suggested reasons that justify the ethicality of euthanasia and knowledge and attitude towards euthanasia. Respondents’ knowledge was assessed with statements that could be answered as “Yes” “No”, or “Don’t know”. Attitude was assessed using positively phrased questions scored on a Likert scale, in response categories of: (1) strongly agree, (2) agree, (3) neutral, (4) disagree, and (5) strongly disagree. Prior to data collection, the questionnaire was pretested on fourth year students and amendments were made accordingly.

### Data processing and analyses

The statistical analyses were done using SPSS version 22. Association between socio-demographic characteristics and participant’s acceptance of euthanasia was checked using logistic regression. *p* value less than 0.05 was considered as statistically significant association.

## Results

### Socio-demographic characteristics of study participants

From 128 questionnaires distributed, 121 returned giving the response rate of 94.5%. Among the returned questionnaire, 4 were excluded during quality check due to their incompleteness and 117 were analyzed. Of the total number of participant, 77 (65.8%) were male of which 40(34.2%) were final year pharmacy students and 106 (90.6%) were in the age range of 20–25 years of which 60 (51.3%) were final year law students. Among the 117 participants, most (58, 49.6%) were Orthodox Christians where law students accounted 34 (29.1%). Regarding with degree of religiosity, 76 (65%) were confirmed that they are active in religious institution of which 40 (34.2%) attributed to pharmacy students (Table 1).

**Table 1:** Socio-demographic characteristics of study participants (n = 117)

Socio-demographic characteristics		Pharmacy (*n* = 55)	Law (*n* = 62)	Total (*n* = 117)
Sex	Male	40 (34.2)	37 (31.6)	77 (65.8)
	Female	15 (12.8)	25 (21.4)	40 (34.2)
	Total	55 (47)	62 (53)	117 (100)
Age category	20–25	46 (39.3)	60 (51.3)	106 (90.6)
	26–30	9 (7.7)	2 (1.7)	11 (9.4)
	Total	55 (47)	62 (53)	117 (100)
Religion	Orthodox	24 (20.5)	34 (29.1)	58 (49.6)
	Muslim	15 (12.8)	18 (15.4)	33 (28.2)
	Protestant	12 (10.3)	8 (6.8)	20 (17.1)
	Others^a^	4 (3.4)	2 (1.7)	6 (5.2)
	Total	55 (47)	62 (53)	117 (100)
Degree of religiosity	Active in religious institution	40 (34.2)	36 (30.8)	76 (65)
	Sometimes attend to religious institution	14 (12)	15 (12.8)	29 (24.8)
	Believer but not participate in religious activities	1 (0.9)	11 (9.4)	12 (10.3)
	Total	55 (47)	62 (53)	117 (100)

^a^Others: Waqefata (3), Adventist (3)

### Knowledge of pharmacy and law final year students on euthanasia

In the current study, 72 (61.5%; 31.6% accounted for law students) of the students were declared that euthanasia is administration of lethal drugs to a patient at the explicit request of that patient. Moreover, majority 87 (74.4%; 41.1% accounted for law students) of the respondents cited that euthanasia is active shortening of the dying process. The participants were also asked that whether euthanasia is legally allowed in Ethiopia and 95(81.2%; 47.9% accounted for law students) affirmed that there is no such legalized practice in Ethiopia. Forty seven (40.2%; 30.8% accounted for law students) stated that there is a difference between active and passive euthanasia according to the laws (Table 2).

**Table 2:** Knowledge of pharmacy and law final year students regarding euthanasia (*n* = 117)

Statements		*n* (%)		
		Yes	No	Don’t know
1. Euthanasia is administration of lethal drugs to a patient at the explicit request of that patient	Pharmacy	35 (29.9)	15 (12.8)	5 (4.3)
	Law	37 (31.6)	10 (8.6)	15 (12.8)
	Total	72 (61.5)	25 (21.4)	20 (17.1)
2. Euthanasia is active shortening of the dying process	Pharmacy	39 (33.3)	12 (10.2)	4 (3.4)
	Law	48 (41.1)	10 (8.6)	4 (3.4)
	Total	87 (74.4)	22 (18.8)	8 (6.8)
3. Euthanasia is physician-assisted suicide	Pharmacy	39 (33.3)	11 (9.4)	5 (4.3)
	Law	54 (46.2)	6 (5.1)	2 (1.7)
	Total	93 (79.5)	17 (14.5)	7 (6)
4. Euthanasia is withholding or withdrawal of treatment	Pharmacy	26 (22.2)	17 (14.5)	12 (10.3)
	Law	43 (36.8)	11 (9.4)	8 (6.8)
	Total	69 (59)	28 (23.9)	20 (17.1)
5. Euthanasia is legally allowed in Ethiopia	Pharmacy	4 (3.4)	39 (33.3)	12 (10.3)
	Law	4 (3.4)	56 (47.9)	2 (1.7)
	Total	8 (6.8)	95 (81.2)	14 (12)
6. Euthanasia is a human act	Pharmacy	27 (23.1)	21 (17.9)	7 (6)
	Law	52 (44.4)	8 (6.8)	2 (1.7)
	Total	79 (67.5)	29 (24.8)	9 (7.7)
7. There a difference between active and passive euthanasia according to the laws	Pharmacy	11 (9.4)	5 (4.3)	39 (33.3)
	Law	36 (30.8)	8 (6.8)	18 (15.4)
	Total	47 (40.2)	13 (11.1)	57 (48.7)

### Attitudes of law and pharmacy final year students towards euthanasia

Concerning the attitudes’ of law and pharmacy final year students towards euthanasia, 40.2% (strongly agree and agree combined) believed that the patient has the right to choose to end his/her own life. Around 45% (strongly agree and agree combined) had the view that euthanasia should be legalized in some circumstances. Notwithstanding of this agreement, only 27.3% (*n* = 32) of the respondents endorsed legalization of euthanasia in Ethiopia (Table 3).

**Table 3:** Attitudes of law and pharmacy final year students towards euthanasia (*n* = 117)

Statements	Field of participants	Responses, *n* (%)					Total, *n* (%)
		1	2	3	4	5	
A patient has the right to choose to end his/her own life	Pharmacy	16 (13.7)	6 (5.1)	11 (9.4)	11 (9.4)	11 (9.4)	55 (47)
	Law	16 (13.7)	9 (7.7)	5 (4.3)	4 (3.4)	28 (23.9)	62 (53)
	Total	32 (27.4)	15 (12.8)	16 (13.7)	15 (12.8)	39 (33.3)	117 (100)
Euthanasia should be legal in some circumstances	Pharmacy	13 (11.1)	15 (12.8)	10 (8.5)	11 (9.4)	6 (5.1)	55 (47)
	Law	11 (9.4)	14 (12)	11 (9.4)	7 (6)	19 (16.2)	62 (53)
	Total	24 (20.5)	29 (24.8)	21 (18)	18 (15.4)	25 (21.3)	117 (100)
The taking of human life is wrong no matter what the circumstances	Pharmacy	21 (18)	7 (6)	12 (10.2)	9 (7.7)	6 (5.1)	55 (47)
	Law	26 (22.2)	17 (14.5)	7 (6)	6 (5.1)	6 (5.1)	62 (53)
	Total	47 (40.2)	24 (20.5)	19 (16.2)	15 (12.8)	12 (10.2)	117 (100)
Patients without hope should not suffer	Pharmacy	11 (9.4)	15 (12.8)	12 (10.2)	10 (8.5)	7 (6)	55 (47)
	Law	18 (15.4)	7 (6)	14 (12)	8 (6.8)	15 (12.8)	62 (53)
	Total	29 (24.8)	22 (18.8)	26 (22.2)	18 (15.4)	22 (18.8)	117 (100)
Patients with a terminal illness should be allowed to die	Pharmacy	7 (6)	6 (5.1)	15 (12.8)	15 (12.8)	12 (10.2)	55 (47)
	Law	14 (12)	6 (5.1)	9 (7.7)	18 (15.4)	15 (12.8)	62 (53)
	Total	21 (18)	12 (10.2)	24 (20.5)	33 (28.2)	27 (23)	117 (100)
Euthanasia should be accepted in today’s society	Pharmacy	6 (5.1)	7 (6)	17 (14.5)	9 (7.7)	16 (13.7)	55 (47)
	Law	7 (6)	1 (0.8)	14 (12)	14 (12)	26 (22.2)	62 (53)
	Total	13 (11.1)	8 (6.8)	31 (26.5)	23 (19.7)	42 (35.9)	117 (100)
Euthanasia should be against the law	Pharmacy	7 (6)	7 (6)	21 (18)	10 (8.5)	10 (8.5)	55 (47)
	Law	29 (24.8)	6 (5.1)	9 (7.7)	9 (7.7)	9 (7.7)	62 (53)
	Total	36 (30.8)	13 (11.1)	30 (25.6)	19 (16.2)	19 (16.2)	117 (100)
Euthanasia gives a person a chance to die with dignity	Pharmacy	12 (10.2)	10 (8.5)	9 (7.7)	7 (6)	17 (14.5)	55 (47)
	Law	8 (6.8)	4 (3.4)	13 (11.1)	18 (15.4)	19 (16.2)	62 (53)
	Total	20 (17)	14 (12)	22 (18.8)	25 (21.4)	36 (30.8)	117 (100)
Euthanasia is acceptable if the person is old	Pharmacy	5 (4.3)	4 (3.4)	13 (11.1)	13 (11.1)	20 (17)	55 (47)
	Law	8 (6.8)	1 (0.8)	7 (6)	15 (12.8)	31 (26.5)	62 (53)
	Total	13 (11.1)	5 (4.3)	20 (17)	28 (24)	51 (43.6)	117 (100)
Ethiopia legislation should be changed to permit euthanasia under certain circumstances	Pharmacy	5 (4.3)	11 (9.4)	14 (12)	10 (8.5)	15 (12.8)	55 (47)
	Law	7 (6)	9 (7.6)	4 (3.4)	10 (8.5)	32 (27.4)	62 (53)
	Total	12 (10.3)	20 (17)	18 (15.4)	20 (17)	47 (40.2)	117 (100)

1 = strongly agree, 2 = agree, 3 = neutral, 4 = disagree, 5 = strongly disagree

### Perceived situations that appropriate for euthanasia and legal status of euthanasia

**Fig. 1:**
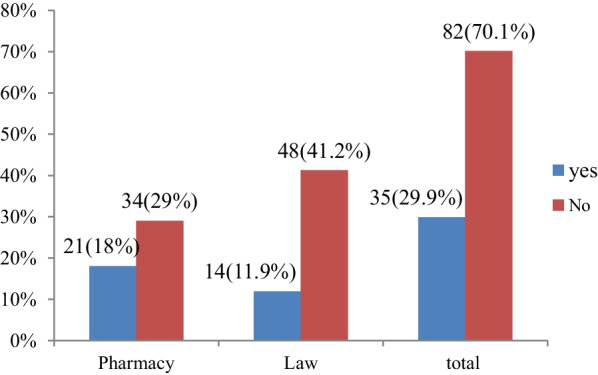
Do you accept euthanasia should be performed? (*n* = 117)

Participants were asked whether or not euthanasia should be performed. Out of all participants (*n* = 117), 35 (29.9%) said euthanasia should be performed and 82 (70.1%) said euthanasia should not be performed (Fig. 1).

**Fig. 2:**
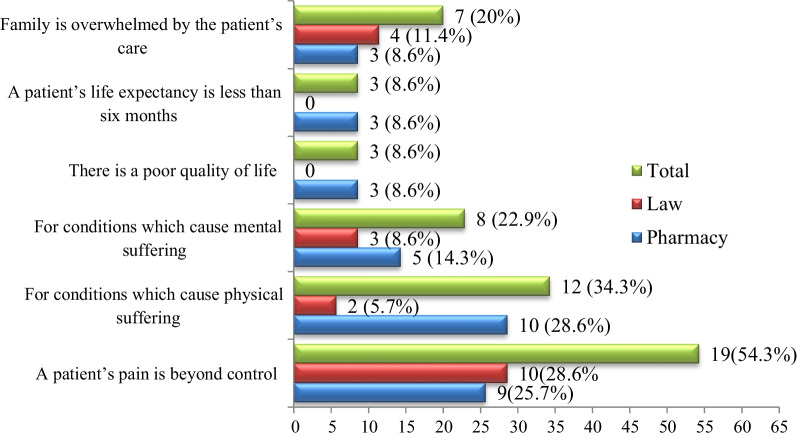
Under what condition do you think that euthanasia should be performed? (*n* = 35)

**Fig. 3:**
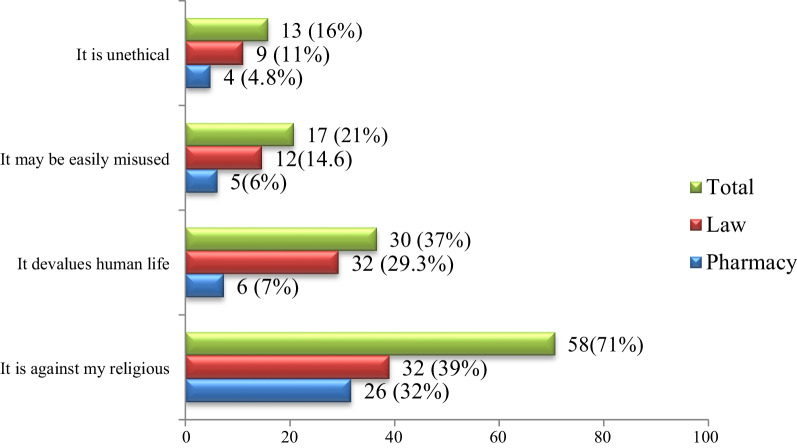
Reason why euthanasia should not be performed (*n* = 82)

The participants who supported performance of euthanasia (*n* = 35) cited conditions under which euthanasia should be performed. Accordingly, 19 (54.3%) of them claimed that when patient’s pain is beyond control and 12 (34.3%) said for conditions which cause physical suffering (Fig. 2). On the other hand, those participants against euthanasia were tested their reason for not supporting euthanasia. In view of that, 58 (49.6%) of them noted that it is against their religion/culture and 30 (25.6%) worried it may devalues human life (Fig. 3).

Participants also questioned “who should decide euthanasia” and the answer for 42.7% (*n* = 50) of them was the patient, whereas 36% replied “the entire physician, patient and the family.” Moreover, 75 (64.1%) of the respondent understood euthanasia is considered as deliberate murder according to laws in Force (Table 4).

**Table 4:** Person who decides euthanasia and euthanasia from legal point of view

Statements		*n* (%)		
		Pharmacy	Law	Total
Who do you think should decide euthanasia	Patient	27 (23)	23 (19.7)	50 (42.7)
	All the physician, the patient and the family	21 (18)	21 (18)	42 (36)
	Family	4 (3.4)	6 (5.1)	10 (8.5)
	Religious leader	2 (1.7)	8 (6.8)	10 (8.5)
	Lawyer appointed by the patient	0 (0)	4 (3.4)	4 (3.4)
	Physician	2 (1.7)	1 (0.9)	3 (2.6)
How euthanasia is considered according to Laws in Force?	Deliberate murder	31 (26.5)	44 (37.6)	75 (64.1)
	No punishment	6 (5.1)	5 (4.3)	11 (9.4)
	Negligence	8 (6.8)	3 (2.6)	11 (9.4)
	Abuse	6 (5.1)	3 (2.6)	9 (7.7)

### Predictors of acceptance of euthanasia

Binary logistic regression analysis was performed to assess the impact of the socio-demographics on acceptance of euthanasia. There were no significant association between age (*p* = 0.549), sex (*p* = 0.951) and degree of religiosity (*p* = 0.083) of respondents on acceptance of euthanasia. Nevertheless, the field of study and religion of participants had significant association with the acceptance of euthanasia. Variables with a *p*-value of < 0.25 were taken to multivariate analysis. Accordingly, the acceptance of euthanasia 3.49 time greater for pharmacy students compared to law students [adjusted odds ratio (AOR) = 3.490; 95% CI 1.346–9.049; *p* = 0.010]. Similarly, the odds for acceptance were lower for Muslim compared to Orthodox students (AOR = 0.186; 95% CI 0.044–0.783; *p* = 0.022) (Table 5).

**Table 5:** Multivariate logistic regression for predictors of acceptance of euthanasia (*n* = 117)

Respondents socio-demographic characteristics		Acceptance of euthanasia		AOR (95% CI)	*p* value
		Yes, *n* (%)	No, *n* (%)		
Field	Law	14 (11.9)	48 (41.2)	1	
	Pharmacy	21 (18)	34 (29)	3.490 (1.346–9.049)	0.01
Religion	Orthodox	24 (20.5)	34 (29)	1	
	Muslim	6 (5.1)	27 (23)	0.186 (0.044–0.783)	0.022
	Protestant	3 (2.6)	17 (14.5)	0.393 (0.029–5.257)	0.480
	Others^a^	2 (1.7)	4 (3.4)	0.396 (.032–4.866)	0.469

^a^Others: Waqefata (3), Adventist (3)

## Discussion

We conducted the present study to assess the knowledge and attitudes of final year students who came from different disciplines, i.e., pharmacy and law, with the intention that they will have a decision power to euthanasia in future.

The current study revealed that nearly 61% of the students responded with declaration that euthanasia is administration of lethal drugs to a patient at the explicit request of that patient. Of those, 31.6% of the response was emanated from final year law students while the remaining went to final year pharmacy students. In addition, large proportion of participants in our study knew that euthanasia is active shortening of the dying process. High percentage (81.2%; *n* = 95) of the participants were also admitted that there is no legal permission of euthanasia in Ethiopia. Moreover, the present study showed that, most of the law students knew the non-existence of the law on euthanasia. This finding is consistent with the finding of study conducted in Brussels, Belgium [1] where 94% of law students knew the existence of legal permission of euthanasia in their country. However, only around 40% of the respondents claimed that there is a difference between active and passive euthanasia.

Regarding the attitudes’ of law and pharmacy final year students towards euthanasia, 40.2% (strongly agree and agree combined) believed that the patient has the right to choose to end his/her own life. This figure is slightly lower than the figure reported elsewhere (68%) [12]. Besides this, around 45% (strongly agree and agree combined) had the view that euthanasia should be legalized in some circumstances. Despite of this agreement, only 27.3% (*n* = 32) of the respondents endorsed legalization of euthanasia in Ethiopia. This finding is comparable with finding of studies from Sudan (23.4%) [24] and Malaysia [22] where 32% of medical students support legalization of euthanasia. In contrary to this, studies from South Africa [3] (43% of medical students), Karachi, Pakistan (61.6% of medical and 58.3% of non-medical students) [8], and Serbia (54% of medical students) [25] were in favor of legalization of euthanasia. The discrepancy might be attributed to cultural difference between the countries and the sample size of study participants.

Less than one-third (29.9%, *n* = 35) of participants in our study favored practice of euthanasia. Comparable results was reported from of study conducted in Sudan (23.4%, *n* = 33) [24], whereas the present figure is higher than the finding of study from Saudi Arabia [15]. Our findings were also in line with study conducted on Turkish health professionals [26] where 33.6% of the respondents favor euthanasia. There are also other studies from Iran University of Medical Sciences (45% of the nursing students) [5], Uppsala University, Sweden (49.4% of law students, 26.8% of medicine students, 28.3% of nursing students) [27], and 60.5% of medical students [28] had positive response toward acceptance of euthanasia.

On the other hand, the general opposition (70.1%) to the practice of euthanasia among the respondents in current study is expected. Similar studies from Sudan [24] and Jahrom University of Medical Sciences, Iran [29], were also reported that most of the final year medical students and paramedical students were against performance euthanasia.

In the present study, most (54.3%; *n* = 19) of the respondents who supported euthanasia cited that euthanasia should be performed in a situation when a patient’s pain is beyond control. However, in study conducted elsewhere [26], conditions which cause physical suffering (17.4%) and mental suffering (11.9%) were the two most common reasons reported by respondents.

Those respondents who opposed euthanasia in the present study (70.1%) stated their reason of which 71% mentioned euthanasia is against their religion. The finding is comparable with finding of study from Saudi Arabia [15] and Karachi [30] in which most of medical students opposed the practice of euthanasia, mainly for religious reasons. In addition, 37% of the participants had fear of euthanasia might devalue human life, and 21% worried about abuse (misuse) of euthanasia. Similar finding were reported in study conducted in Turkey where 38.4% of study participants were concerned about abuse of euthanasia, 30% mentioned religious reasons and 29.6% found it unethical [26]. Other study from Malaysia also reported that 91% of the respondents were concerned about the misuse of euthanasia among health professionals if it were to be legalized [22].

Approximately, 43% of participants believed that the decision for the implementation of euthanasia should be made by the patient. This finding may supported by the figure reported in other study (40%) [12]. Study from Turkey [26], however, reported higher percentage respondents (73.3%) where the senior nursing students claimed that patient him/herself should decide euthanasia. This discrepancy might be attributed to different sample size, mode of data collection and field of study. Other possibilities, such as patients’ relatives (families), religious leader and lawyers, were overlooked by most of the responders in our study. On the other hand, three-fourths of the respondent in the current study considered euthanasia as deliberate murder. This finding was supported by finding of similar study in Turkey in which more than half of the respondents mentioned euthanasia as deliberate murder [26].

Regarding predictors of acceptance of euthanasia, the acceptance of euthanasia were greater among pharmacy students compared law students. This might be attributed to health science students encounter desperation of people during internships or clerkship at hospitals and the experience that they gained causes them to find euthanasia favorable. Moreover, the acceptance of euthanasia was lowers in Muslim students as compared to orthodox students. This finding is consistent with the finding of other study where the odds of accepting euthanasia for medical students were greater than for students of philosophy and positive attitude toward euthanasia was less likely among Muslim students compared to Catholic students [1].

## Limitation

Since a self-administered questionnaire was used, the response bias is likely and the associated biases are acknowledged. In addition, due to the relatively small sample size, and single site, the generalization of the results is limited.

## Conclusions

The final year law and pharmacy students were aware of euthanasia. However, majority of students did not reveal favorable attitude toward euthanasia and its acceptance was still low. Participants’ field of study and religion were significantly affect acceptance of euthanasia As the current study is limited to pharmacy and law students, the authors suggest that future studies should involve various healthcare practitioners, lawyers, students from different disciplines, patients and patients’ family and general population at large to investigate more about euthanasia in Ethiopia.

## Data Availability

The data used to support the findings of this study are available from the corresponding author upon reasonable request.
